# Allosteric Site Mediates Inhibition of Tonic NMDA Receptor Activity by Low Dose Ketamine

**DOI:** 10.21203/rs.3.rs-3304783/v1

**Published:** 2023-09-21

**Authors:** Gabriela Popescu, Jamie Abbott, Han Wen, Beiying Liu, Sheila Gupta, Gary Iacobucci, Wenjun Zheng

**Affiliations:** Jacobs School of Medicine and Biomedical Sciences/University at Buffalo, SUNY; Jacobs School of Medicine and Biomedical Sciences/University at Buffalo, SUN; SUNY Buffalo; Jacobs School of Medicine and Biomedical Sciences/University at Buffalo, SUN; University at Buffalo

## Abstract

Ketamine, a general anesthetic, has rapid and sustained antidepressant effects when administered at lower doses. At anesthetic doses, ketamine causes a drastic reduction in excitatory transmission by lodging in the centrally located hydrophilic pore of the NMDA receptor, where it blocks ionic flow. In contrast, the molecular and cellular targets responsible for the antidepressant effects of ketamine remain controversial. Here, we report functional and structural evidence that, at nanomolar concentrations, ketamine interacts with membrane-accessible hydrophobic sites where it stabilizes desensitized receptors to cause an incomplete, voltage- and pH-dependent reduction in NMDA receptor activity. This allosteric mechanism spares brief receptor activations and reduces preferentially currents from tonically active receptors. The hydrophobic site is a promising target for safe and effective therapies against acute and chronic neurodegeneration.

## One sentence summary

Ketamine acts at two chemically discrete sites on NMDA receptors to reduce currents with distinct potencies and mechanisms.

## INTRODUCTION

Ketamine is a synthetic drug with a complex spectrum of clinical effects. At high plasma concentrations (5–10 μM), it induces general anesthesia (1), by specifically blocking excitatory synaptic transmission mediated by NMDA receptors (2). The reported half-maximal effective concentration of ketamine on recombinant NMDA receptor currents varies with the concentration and duration of agonist application, with transmembrane voltage and external pH, Mg^2+^, and Ca^2+^ concentrations (3–6). These and other functional observations support the hypothesis that at anesthetic concentrations, ketamine reduces excitatory transmission by binding deep inside the membrane-embedded pore of open NMDA receptors, where it blocks ionic flux (7). Structural studies validated this mechanism and identified pore-lining residues that interact directly with ketamine and mediate its potency (8, 9).

At subanesthetic plasma concentrations (0.35–0.85 μM), ketamine has rapid anti-depressant, analgesic, and anti-inflammatory effects (1, 10). The mechanism by which the clinical benefits of low-dose ketamine occur is unknown. The anti-depressant effect is of special interest because depression is one of the most prevalent mood disorders and a leading cause of disability globally. Moreover, existing therapies take weeks to work and remain ineffective for most patients (11). In contrast, the antidepressant effect of low-dose ketamine is apparent within hours, can last for up to a week and can resolve treatment-resistant symptoms and suicidal ideation (10–12). Multiple lines of evidence implicate NMDA receptors as the molecular target for the clinical effects of low-dose ketamine, however this remains controversial (1, 13, 14). Among the facts casting doubt is a lack of correlation between the doses at which it is effective as antidepressant and as an open channel blocker. More puzzling are reports that memantine, a drug that blocks NMDA receptor currents with potency, kinetics, and mechanism very similar to those of ketamine, has no anti-depressive effects (15). Lastly, of the two ketamine enantiomers, although the S(−) form is slightly more potent at blocking NMDA receptor currents, the R(+) form is a more potent anti-depressant (16). For this reason, several alternative mechanisms for the anti-depressant effects of ketamine are being actively investigated.

Despite its abundance, the literature documenting the effects of ketamine on NMDA receptor currents is surprisingly sparse in the sub-micromolar range of the dose-response relationship. Moreover, due to considerable variations in the experimental conditions used across laboratories, the values reported for the ketamine EC_50_ vary widely (0.3–2.4 μM) (3, 4, 6). To gain clarity, we optimized experimental conditions and reexamined the dose-response relationship over an extended range of concentrations.

## RESULTS

### Complex dose-response relationship for KET inhibition of NMDA receptor currents

**Figure 1:**
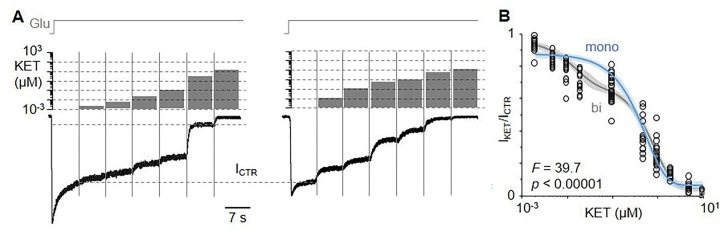
Extended Dose Response Relationship. (A) Whole-cell current traces elicited with glutamate (Glu) and with two series of ketamine (KET) concentrations, from GluN1-1a/GluN2A (WT) receptors expressed in HEK293 cells, are shown normalized to the control steady-state current level (I_CTR_). (B) Summary of pooled results (black circles, n = 24 cells, n >12 cells per concentration) and fitted mono- (blue) and bi-phasic (grey) dose-response functions (solid lines) with associated 95% confidence intervals (shaded). Indicated F and p statistics were calculated as described in Methods.

We measured the effects of racemic (R, S)-ketamine (KET) on whole-cell currents elicited from recombinant wild-type NMDA receptors (GluN1-1a/GluN2A, WT) with maximally effective agonist concentrations (1 mM Glu, 0.1 mM Gly), and strong depolarization (−100 mV), in low Ca^2+^ (0.25 mM), and physiologic pH (7.2). These conditions balance effects on ketamine potency, which increases with acidic pH and membrane potential (7), with effects on receptor gating, which decreases with acidic pH and Ca^2+^ concentrations (17, 18). We applied KET at increasing concentrations (0.002–10 μM) onto the steady-state phase of the glutamate-elicited current (I_CTR_) and measured the residual current in the presence of KET (I_ket_) (Fig. 1A).

Plotting the fraction of residual current (I_KET_/I_CTR_) as a function of KET concentration revealed a complex relationship (Fig. 1B). Fitting a mono-exponential function to these data returned a potency within the previously reported range: 0.37 ± 0.07 μM (R^2^, 0.91; SSE, 2.28). However, a biphasic function described the data more closely (R^2^, 0.98; SSE, 0.81) In addition to the inflection point recognized previously (0.67 ± 0.15 μM), a biexponential function revealed a new inflection point in the low nanomolar range (0.02 ± 0.01 μM), suggesting a previously unrecognized high-potency inhibitory site (**Supplementary Table 1**).

### Ketamine interacts with two types of binding sites on NMDA receptors.

**Figure 2:**
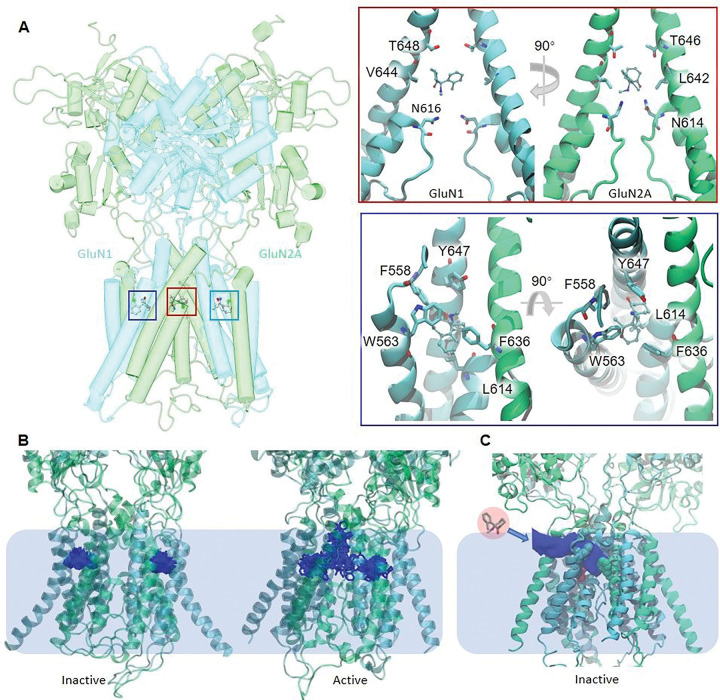
Simulated interactions between S-KET+ and NMDA receptors. (A) Left, results from local docking of protonated S(−)-ketamine onto an inactive NMDA receptor structure lacking C-terminal domains (PDB ID: 6whs) illustrates three putative binding sites (boxed in left panel): one located centrally in the pore (site 1, red) and two symmetry-related intra-membrane sites: site 2 (dark blue) and site 3, (light blue). Right, detailed positioning of S-KET+ in site 1 (top) and in site 2 (bottom) and key contacts with residues in GluN1 (blue) and GluN2A (green) subunits. (B) Results from MD simulation of S-KET+ with inactive (left) and active (right)

To explore the possibility of additional KET-binding sites that are structurally distinct from the central site described previously (8, 9), we performed molecular docking for protonated S(−)-ketamine (S-KET^+^) on four NMDA receptor conformers: two functionally inhibited structures (PDB IDs: 4tlm, 6whs) (19, 20), one semi-active structure (PDB ID: 6wi1) (20), and one open-pore structural model that we built in an earlier study (21). For this exploratory global docking we omitted the extracellular domains from these structures to focus on the transmembrane domains. The results illustrated three putative binding sites: one was centrally located in the aqueous pore, and two symmetry-related but not identical, were located laterally to the pore. Intrigued by these results, we performed more extensive local docking and molecular dynamics (MD) simulation on the four structural templates after omitting their N-terminal domains to reduce computational time (Fig. 2A). Results showed that at the central location (site 1), S-KET^+^ relies on contacts with GluN1 T648, V644, and N616, and with GluN2A T646, L642, and N614, as reported previously for ketamine (8, 9) and other open channel blockers (19, 22) (Fig. 2B, **Supplementary** Fig. 1). At the two membrane-embedded sites (sites 2/3), S-KET^+^ engaged mainly with GluN1 F558, W563, Y647, and L614 and with GluN2A F636 (Fig. 2A and C, and **Supplementary Fig. 1)**.

To examine in more detail the binding energies and dynamics in these three sites, we performed MD simulations using the S-KET^+^-docked receptor structures for all four systems as detailed in **Supplementary Methods**. For the central site, results reveal multiple binding modes, as reported previously (8) (Fig. 2B, and **Supplementary Fig. 2**). In contrast, S-KET^+^ interacts more stably with the lateral sites in the inactive state. In the semi-active and the open-pore active state, S-KET + is more dynamic and can slide into the pore to reach the central site. Differential binding to distinct functional states is a strong indicator of a gating modifier, suggesting that the lateral sites mediate an allosteric mechanism of receptor inhibition.

Based on our results and the present literature, we hypothesized that ketamine may access the lateral sites through a membrane accessible path to stabilize receptors in closed states. We modeled the binding pathway and explored the communication between the allosteric site and the central pore with steered MD simulations of the inactive and the active structures by applying a pulling force to drive the S-KET^+^ away from the allosteric site. In both simulations we observed that S-KET^+^ diffused into the membrane following the lateral tunnel described previously (22) (Fig. 2B and **Supplementary Movie S1**). In addition, in the active state simulation, S-KET^+^ also diffused into the central pore. (**Supplementary Movie S2**).

To test the hypothesis that GluN2A-F636 contributes specifically to a lateral binding site, we examined the effect of the GluN2A F to L substitution on S-KET^+^ binding in the inactive receptor conformation. MD simulations revealed greater fluctuations of S-KET^+^ relative to the wild-type complex, suggesting that direct interactions between GluN2A-F636 and S-KET^+^ contribute to ligand stability in this lateral site (**Supplementary Tables 2 and 3).**

Taken together, the docking and MD simulations suggest that S-KET^+^ interacts with a previously unrecognized lateral site within the transmembrane domain of NMDA receptors, which differs from the centrally located site in three key aspects: it relies on contacts with distinct, mostly aromatic residues (GluN1-Y647 and GluN2A-F636); it may be accessed from the membrane through a lateral tunnel; and it may reduce NMDA receptor currents through an allosteric rather than an open-channel block mechanism. Next, we aimed to test these important predictions.

### Residues in the aromatic site modify the nanomolar region of the extended dose-response relationship.

**Figure 3:**
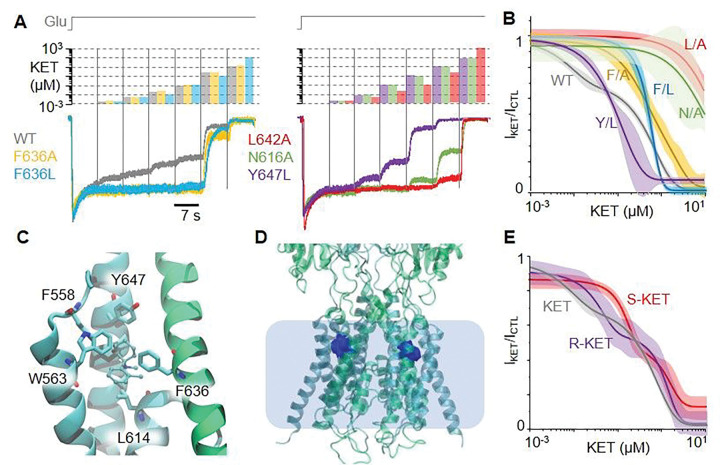
Probing putative contacts between KET and NMDA receptors. (A) Whole-cell Na+ currents elicited with Glu (1 mM) at pH 7.2 and −100 mV from cells expressing WT or mutated NMDA receptors, with the indicated series of KET concentrations. (B) Extended dose response relationships illustrated as best fitting functions (solid lines) with associated 95% confidence intervals (shades area) to pooled data for each receptor (n, 12 – 31 cells per construct, 6 – 15 cells per concentration). (C) Results from MD simulation of protonated R(+)-ketamine onto an inactive receptor conformation illustrates principal contacts with residues in site 2. (D) Results from MD simulation of R-KET+ with inactive NMDA receptor structures illustrates its trajectories (blue). (E) Extended dose-response relationships for WT receptors with ketamine isoforms are illustrated as best fits of biphasic models (lines) and 95% confidence intervals (shaded area) of pooled results from whole-cell Na+ currents recorded at pH 7.2 and −100 mV for each ketamine preparation. n = 8 – 11 cells per enantiomer, 4 – 11 cells per concentration.

We constructed extended dose-response relationships, as described above, for a series of GluN1/GluN2A receptors that had single-residue substitutions at sites predicted to interact with site 1, site 2, or both sites (Fig. 3A). Results show that modifying the side chains of residues that are specific to sites 2/3, such as GluN1 Y647 (Y/L) and GluN2A F636 (F/A and F/L) changed the dose-response relationship in the low nanomolar range, as expected when perturbing the higher-affinity site. In contrast, modifying side chains of residues that are specific to site 1, such as GluN2A L642 (L/A) or that are common to all three sites, such as GluN1 N616 (N/A) strongly shifted the dose-response function in the micromolar range (Fig. 3B and **Supplementary Table 1)**.

### R-KET + forms stronger contacts with the aromatic site

Presently, experimental structural information is only available for the interaction of NMDA receptors with S-KET^+^. To investigate potential differences in how enantiomers contact NMDA receptor residues, we repeated the local docking and MD simulation at the lateral sites with protonated R(+)-ketamine (R-KET^+^). Results showed largely similar contacts as with S-KET^+^, except that, relative to S-KET^+^, R-KET^+^ formed additional interactions through its aromatic ring (**Supplementary Fig. 1**) and consequently remained more stable along the MD simulation (Fig. 3D and **Supplementary Table 2,3**).

To test the prediction that R-KET + interacts with the aromatic site, more strongly than S-KET+, we constructed extended dose-response relationships for S-KET and R-KET, as described above for racemic ketamine (KET). Results show that the dose response function is left-shifted for R-KET relative to S-KET, especially in the low concentration range, supporting the prediction that R-KET has stronger affinity for the aromatic site than for S-KET (Fig. 3B and **Supplementary Table 1**).

### Ketamine can access its effector sites through a lateral tunnel.

**Figure 4:**
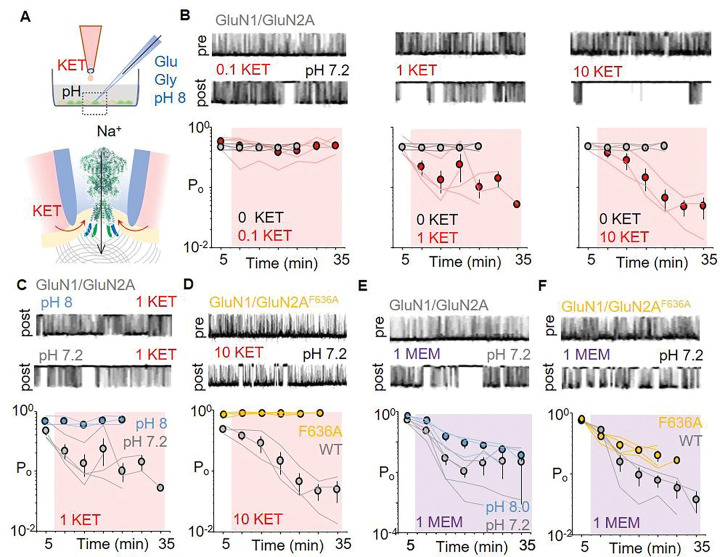
Probing a trans-membrane pathway for drug access to effector sites within NMDA receptors. (A) Cartoon illustrates setup used to record on-cell stationary Na^+^ currents from NMDA receptors isolated within the recording electrode containing agonists (Glu and Gly) at pH 8 (blue). After recording basal activity (pre, unshaded) for 5 min, KET (or MEM) is added to the bath at pH 7.2 (grey) or as indicated, and activity is recorded for another 30 min (post, shaded). (B) Top, Currents recorded from WT receptors before (pre) and after equilibration in KET at the indicated concentrations (post). Bottom, time-dependent change in calculated Po along individual recordings obtained with 0 KET (grey) and the indicated KET concentrations (red). (C) Currents recorded after adding KET (1 μM) in the bath at pH 8 (blue) relative to pH 7.2 (grey). (D) Activity from GluN1/GluN2AF636A (yellow) relative to WT (grey) with 10 μM KET. (E) Activity from WT with 1 μM MEM added to the bath at pH 7.2 (grey) relative to pH 8 (blue). (F) Activity from GluN1/GluN2AF636A (yellow) relative to WT (grey) with 1 μM MEM in the bath at pH 7.2.

To investigate the hypothesis that ketamine can reach its effector sites on NMDA receptors through a membrane-accessible pathway, we leveraged the cell-attached voltage-clamp method, where actively gating receptors can be isolated within the membrane enclosed by the recording pipette through a high-resistance seal (Fig. 4A). In this arrangement, stationary currents can be recorded for extended periods of time from receptors experiencing a constant external milieu, while the contents of the solution bathing the rest of the cell can be manipulated separately. We recorded on-cell Na^+^-only currents from NMDA receptors exposed to saturating concentrations of agonists (1mM Glu and 0.1 mM Gly), and in the absence of blocking divalent cations (1 mM EDTA) and low proton concentrations (pH 8) (23). In patches with a single active receptor, as indicated by the absence of overlapping openings, we recorded basal receptor activity for 5 min, after which we applied KET in the pH 7.2 bath, and recorded activity for another 30 min (Fig. 4B).

For WT receptors, we observed a time- and concentration-dependent reduction in receptor open probability (P_o_) after adding to a pH 7.2 bath KET concentrations of 1 μM and above (Fig. 3B). However, when added to a pH 8 bath, 1 μM KET had no effect on receptor activity (Fig. 3C). KET is a weak base with a reported dissociation constant (pKa) of 8.5. Our result that increasing the pH by 0.6 units reduced the effectiveness of bath-applied KET to inhibit NMDA receptor activity indicates that protonated KET is the active species even when KET can only access its effector site by membrane diffusion.

Although KET is a strongly lipophilic molecule, with reported partition coefficient (logP) of 4.4, the partition coefficient for the active species (protonated ketamine) is more difficult to ascertain and is likely voltage dependent. Therefore, the results from on-cell recordings can be compared only qualitatively with those obtained with whole-cell recordings, due to differences in membrane distribution for protonated ketamine, caused by (at least) differences in the voltage driving its diffusion (−100 mV for whole-cell, and approx. −10 mV for cell-attached). Nonetheless, the result that GluN1/GluN2A(F636A) receptors were insensitive to anesthetic concentrations of KET (10 μM) at physiologic pH (7.2) demonstrates a critical role for GluN2A-F636 in the inhibitory potency of bath-applied KET (Fig. 3D).

### GluN2A-F636 is required for ketamine, but not memantine, access through the membrane.

In contrast to the results obtained with bath-applied KET, experiments with bath-applied memantine (MEM), show that the receptor sensitivity to bath-applied MEM is largely independent of the bath pH (Fig. 3E). This result likely reflects the stronger base character of MEM (pKa = 10). However, the observation that receptor sensitivity to bath-applied MEM is largely independent of GluN2A-F636 reveals an unsuspected mechanistic difference in the actions of KET and MEM on NMDA receptor currents (Fig. 3F). It demonstrates that MEM inhibition is independent from the aromatic site identified here as critical for KET, and therefore, the allosteric site is specific for KET and may be responsible for the unique clinical effects of low-dose ketamine.

### Low-dose ketamine stabilizes desensitized NMDA receptors.

Upon visual inspection, the mechanism by which high concentrations (10 μM) of bath-applied KET (at pH 7.2) reduced the activity of GluN2A-F636A (F/A) receptors (monitored at pH 8), appeared phenotypically different from a similar level of inhibition observed for WT receptors (by 1 μM). Traces from F/A had short interruptions within bursts of activity, as expected for open-channel block, whereas traces from WT had longer silent periods between bursts of activity, as expected from a mechanism that increased desensitization. We explored this observation by analyzing the bursting behavior in single-channel activity (**Supplementary Fig. 3**). These analyses show that upon bath application of KET, the P_o_ in recordings obtained from WT, F/A, and F/L receptors decreased with KET concentration, consistent with results from whole cell currents (Fig. 3B). However, the P_o_ within bursts was insensitive to KET concentration, such that the decrease in activity could be explained entirely by changes in burst duration and frequency. This behavior suggests that when applied in the bath, KET reduces receptor activity mainly by changing its desensitization kinetics.

**Figure 5:**
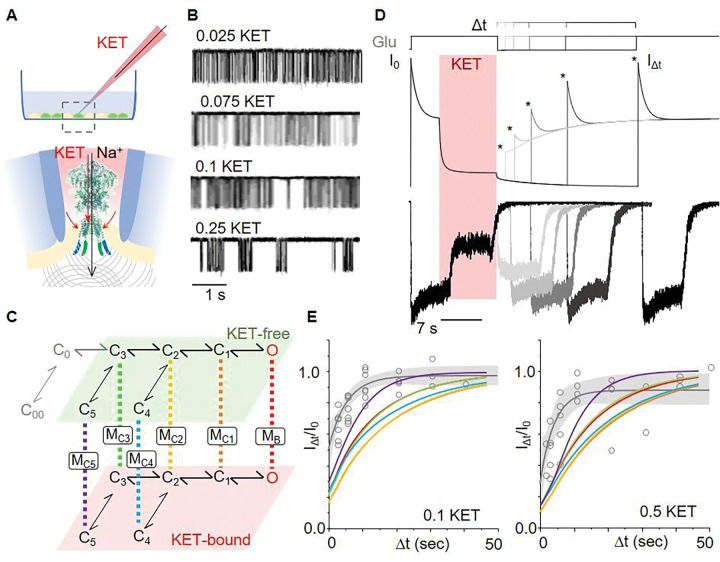
Probing the inhibitory mechanism of low-dose ketamine. (A) Cartoon of the setup used to record on-cell stationary Na^+^ currents from NMDA receptors isolated within a membrane patch exposed to agonists (Glu and Gly) and KET, at pH 7.2, and +100 mV. KET can access its binding sites through the open pore, together with Na^+^, and through the pipette-enclosed membrane-patch. (B) Inward Na^+^ currents recorded from individual receptors with the indicated sub-micromolar concentrations of KET. (C) Kinetic model used to fit the sequence of closed and open intervals observed in single-channel recordings includes gating transitions among agonist-bound closed (C1-C5) and open (O) kinetic states that are KET-free (top tier) and KET-bound (bottom tier), respectively. Two glutamate-binding steps initiate the activation of resting (C_00_ and C_0_) receptors, and a KET-binding step connects the two tiers. Six hypothetical models (M_C1_-M_C5_, and M_B_) differ in the kinetic state (C_1_-C_5_, or O) proposed to allow transition between tiers with highest probability. (D) Schematic of a three-pulse stimulation protocol illustrates the timing and duration of consecutive Glu-KET-Glu applications. Top traces (thin lines) represent the macroscopic current simulated with model M_C5_. Bottom traces (thick lines) illustrate whole-cell currents recorded experimentally. (E) Summary of results illustrates the increase in current response to the second glutamate pulse (I_Dt_) with time (Dt), as predicted (colored lines) with the models in panel D, and as measured experimentally (circles) with fitted exponential function (grey line).

To investigate the hypothesis that the high-affinity site reduces receptor currents by changing channel gating rather by blocking the pore, we examined the gating kinetics of currents obtained from cell-attached patches containing a single active wild-type NMDA receptor, with low concentrations of KET included in the recording pipette, at physiologic pH (7.2) (Fig. 5A). We obtained high quality recordings with several sub micromolar concentrations of KET: 0.025 μM (n = 2), 0.075 μM (n = 1), 0.10 μM (n = 2), and 0.25 μM (n = 2) (Fig. 5B, and **Supplementary Tables 4 and 5**) and used these for kinetic modeling across concentrations.

In the conditions used here, NMDA receptors activate by transitioning along a path that connects resting states (C_0_, C_00_) with open (O) states, through three pre-open closed states (C_1_-C_3_). In addition, this activation path is interrupted by infrequent transitions into long closed states (C_4_, C_5_), which represent desensitized receptors (24). To account for KET-bound states we developed a tiered model in which the top tier represents gating in the absence of KET and the bottom tier represents gating when bound with KET. Receptors were allowed to transition between tiers via one of six possible pathways, resulting in six distinct models (Fig. 5C; **Supplementary Table 5**). We fitted globally each of these six models to the pooled single-channel data obtained at low KET concentrations and optimized rate constants for each. Next we used the models with optimized rate constants to envision several stimulation paradigms that would allow us to rank these models with respect to their probability of accurately representing receptor behavior.

Among several protocols that displayed reasonable discrimination between the macroscopic behavior of currents simulated with the six tiered-models, we selected for experimental testing the following, Glu-KET-Δt-Glu, protocol. In this protocol, receptors are stimulated with a pulse of Glu, after which KET is applied onto the steady-state phase of the current. Next, both Glu and KET are washed for a variable amount of time (Δt), after which current recovery is tested with a last pulse of Glu (Fig. 5D). Results show that at sub-micromolar concentrations of KET, a model where KET binds preferentially to state C_5_ (M_C5_) predicts current recoveries that are closest to those recorded experimentally. Taken together, these results show that low concentrations of ketamine reduce NMDA receptor currents with an allosteric mechanism that increases the desensitization of tonically active receptors.

## DISCUSSION

Our results reveal that ketamine exerts a dual inhibitory effect on NMDA receptor-mediated currents. As described previously, protonated ketamine reduces currents with an open pore-block mechanism with potency in the micromolar range of concentrations. Here, we demonstrate that at sub-micromolar concentrations, ketamine acts with an allosteric mechanism to stabilize desensitized receptor states. The specific effects of low-dose ketamine went unnoticed in previous studies likely due insufficient experimental sampling for a preparation consisting of multiple molecular forms for both ketamine and NMDA receptors. Additional confounding variability may have arisen from the dual, hydrophilic and hydrophobic, access pathways to the two distinct effector sites.

Ketamine is a small organic molecule, which has a chiral center, amphipathic character, and behaves as a weak base in aqueous solutions. It is usually supplied commercially as a racemic mixture of R(+) and S(−) enantiomers, which exist as a dynamic equilibrium of protonated and neutral forms. Each of these four molecular forms distributes in biological membranes in proportions that are dependent on the pH of the aqueous solution, the lipid composition of the membrane, and the trans-membrane voltage. Our results show that the protonated enantiomers, which are the active ingredients in this mixture, bind to NMDA receptors at two separate sites with distinct affinities. Lastly, the sites themselves change their accessibility to and affinity for ketamine during the natural activation cycle of the receptor, such that conditions that affect receptor gating, such as pH, Ca^2+^, Zn^2+^, and the concentration and the duration of exposure to agonists (Glu and Gly or D-Ser) will also affect the observed dose-response relationship.

Our results are consistent with a large body of literature demonstrating that the anesthetic effects of micromolar ketamine occur due to its ability to bind in the central pore of NMDA receptors, where it occludes current flow through open NMDA receptors. This open pore-block mechanism is voltage- and use-dependent; it is more sensitive to the S-KET^+^ enantiomer and relies heavily on GluN2A-L642, which can be accessed rapidly through the aqueous pore when receptors are open. In contrast, we demonstrate that at sub-micromolar concentrations, ketamine reduces NMDA receptor gating by stabilizing a desensitized receptor conformation. This effect is mediated by a previously unsuspected site located in the transmembrane domain, laterally and adjacent to the central pore. It consists of aromatic residues such as GluN1-Y647 and GluN2A-F636, and it is more sensitive to the R-KET^+^ enantiomer. Ketamine can access this site through a lateral tunnel regardless of receptor activation; however, like the pore block mechanism, this allosteric inhibitory effect is also use- and voltage-dependent and becomes apparent only after receptors desensitize. These new observations strongly support the hypothesis that the hydrophobic site we describe here mediates the anti-depressive effects of ketamine. In this context, our result that GluN2A-F636 is required for inhibition by bath-applied KET, but not MEM, provides a simple explanation for why MEM has no anti-depressive effects despite its similarity with KET in blocking the NMDA receptor pore.

Importantly, the allosteric mechanism and membrane-access pathway we describe here for NMDA receptor inhibition by low concentrations of ketamine can explain a growing body of evidence documenting cell-specific and patient-specific effects of low-dose ketamine. First, because the inhibitory effect of low-dose ketamine becomes apparent only after receptors desensitize, it will target tonically active NMDA receptors, such as those expressed at extrasynaptic sites. Depending on experimental conditions, this inhibition may be observed *in vitro* as a reduction in spontaneous glutamate release by pyramidal neurons (25) or as a hyperpolarization of GABA-ergic interneurons (26). Second, because the effective species is the protonated KET, the effect of low-dose KET will depend on the interstitial pH, which can vary between individuals. Lastly, because the allosteric site is accessed through the membrane, the effect of low-dose KET will depend on the membrane composition, structure, and transmembrane voltage surrounding the targeted NMDA receptors.

Therefore, the clinical effect of KET will depend not only on the dose, route of administration, and type of KET (R or S) administered, but also on body composition, existing circuits, and level of arousal of each patient. Our detailed description of a high-affinity site, unique mechanism, and distinct access pathway represent a strong springboard for the development of more effective interventions to correct neuropsychiatric disorders. It guides further research in the expression and mechanism of NMDA receptors and paves the way for developing tailored treatment regimens for each patient. Moreover, because drugs active at the hydrophobic site we describe here cause incomplete NMDA receptor inhibition and because they only affect responses of tonically active receptors, it can be targeted to reduce excitotoxicity in acute and chronic neurodegenerative conditions.

## Data Availability

Requests for further information, resources, or reagents should be directed to and will be fulfilled by the senior author (GKP).
